# Are Freshwater Mixotrophic Ciliates Less Sensitive to Solar Ultraviolet Radiation than Heterotrophic Ones?^1^

**DOI:** 10.1111/j.1550-7408.2011.00540.x

**Published:** 2011-05

**Authors:** BETTINA SONNTAG, MONIKA SUMMERER, RUBEN SOMMARUGA

**Affiliations:** Laboratory of Aquatic Photobiology and Plankton Ecology, Institute of Ecology, University of InnsbruckTechnikerstr. 25, Innsbruck, Austria

**Keywords:** *Chlorella*, lake, mycosporine-like amino acids, plankton, symbiosis

## Abstract

We tested whether mixotrophic ciliates are more resistant to solar ultraviolet radiation (UVR) than heterotrophic ones because symbiotic algae can provide self-shading by cell matter absorption and eventually by direct UV screening from mycosporine-like amino acids (MAAs). Sensitivity of a natural assemblage to solar radiation was tested in experiments in the original lake and in a more UV transparent alpine lake after transplantation of the ciliates. In both lakes, the assemblage was exposed either to full sunlight, to photosynthetically active radiation only, or kept in the dark. In each lake, exposure was for 5 h at the surface and at the depth corresponding to the 10% attenuation depth at 320 nm. Overall, when the assemblage was exposed to surface UVR, only one out of four dominant mixotrophic ciliates, *Vorticella chlorellata*, was more resistant than heterotrophic species. The higher UV resistance in *V. chlorellata* was related to the presence of MAAs and the high percentage of ciliate volume occupied by algal symbionts. Our results indicate that effects of UVR were species-specific and depended on efficient screening of these wavelengths, but also on the depth preference of the ciliates and thus, on their previous exposure history to UVR.

THROUGHOUT the year, a succession of distinct ciliate assemblages can be observed in temperate lowland lakes (Weisse and Müller 1998). Especially in the epilimnion, ciliate assemblages respond immediately to changes in temperature or in food availability such as those caused by algal blooms (Müller et al. 1991; Weisse et al. 1990). For example, algivorous prostomatid and oligotrich species such as *Balanion planctonicum* and urotrichs usually dominate during the phytoplankton spring bloom and are then followed by a highly diverse community of many co-existing species (Müller et al. 1991; Sonntag et al. 2006). Besides net heterotrophic species, many mixotrophic ciliates that either bear algal symbionts or sequester algal plastids (kleptoplasts) prevail during the summer months in surface waters where they can account for more than 50% of the total ciliate abundance (e.g. Carrias, Amblard, and Bourdier 1998; Dolan 1992; Müller et al. 1991; Pace 1982; Sonntag et al. 2006). Living in sunlit waters allows mixotrophic ciliates and their symbionts to optimally use nutrients and light (Modenutti et al. 2005; Wölfl and Geller 2002). This mutualistic relationship is primarily considered as a nutritional advantage and adaptation to live in oligotrophic environments (Dolan and Pérez 2000). However, exploiting the upper layers of the water column also implies that the ciliates are exposed to damaging levels of incident ultraviolet radiation (UVR, 280–400 nm).

Ultraviolet radiation is known to damage DNA and cell compartments, but organisms have developed manifold strategies of avoidance, protection, or repair. Planktonic organisms, for example, carry out vertical downward migrations in the water column to escape high irradiation intensities (e.g. Leech and Williamson 2001) or they may accumulate photoprotective compounds. The importance of UVR as a natural stress factor for mixotrophic ciliates is reflected in the recent finding that *Chlorella*-bearing species are able to use secondary metabolites known as mycosporine-like amino acids (MAAs) for their photoprotection (Sonntag, Summerer, and Sommaruga 2007; Summerer, Sonntag, and Sommaruga 2008). Even in the absence of MAAs, algal symbionts such as *Chlorella* can reduce damage of sensitive cell compartments by self-shading (Sommaruga and Sonntag 2009; Summerer et al. 2009). Consequently, the mutualistic relationship between ciliates and their symbiotic algae should be considered not only from a nutritional, but also from a photobiological perspective (Sommaruga and Sonntag 2009). Yet, these recent findings were obtained in experiments with *Paramecium bursaria* and there is little information on how freshwater euplanktonic species respond to UVR. In particular, the total number of symbiotic *Chlorella* differs among ciliates, as well as their arrangement and volume occupied in the host. Therefore, we may expect to find different tolerance among mixotrophic species.

Only a few studies have addressed how different ciliate species respond to UVR, and those results available indicate that sensitivity is species-specific (Giese et al. 1965; Mostajir et al. 1999; Sanders et al. 2005; Sommaruga et al. 1999; Sonntag, Summerer, and Sommaruga 2011; Wickham and Carstens 1998). For example, Sanders et al. (2005) tested the UV sensitivity of two ciliate species from an oligotrophic lake. After exposure to UV-B (280–315 nm) and photoreactivating radiation (UV-A, 315–400 nm and visible light, 400–700 nm), *Glaucoma* sp. was able to photorepair, whereas *Cyclidium* sp. was not and died. Indirect effects of UVR on the structure and the dynamics of planktonic food webs have also been observed (Mostajir et al. 1999; Wickham and Carstens 1998). In a mesocosm experiment, Mostajir et al. (1999) found a strong decline of predatory ciliates under UV-B stress leading to a positive feedback on their prey.

In terms of UV transparency, there are large differences between subalpine and alpine lakes, i.e. located above the treeline (Rose et al. 2009). In subalpine lakes, UVR is strongly attenuated within the uppermost meters of the mixed layer and therefore, UV sensitive species may remain below the highest UVR levels close to the surface (Rose et al. 2009). By contrast, organisms in alpine lakes are exposed to elevated UVR levels throughout the water column, mainly because of the low concentration in chromophoric dissolved organic matter that acts as an external filter and of the higher incident UVR, particularly in the UV-B range, at higher elevations (Sommaruga and Psenner 1997).

In the subalpine lake Piburgersee (PIB), the ciliate assemblage found in July is characteristically dominated by mixotrophic, mainly *Chlorella*-bearing ciliate species with maxima of distribution in the uppermost meters of the water column. Therefore, we selected this lake to test the idea that mixotrophic ciliate species are more resistant against solar UVR than heterotrophic ones. For this objective, we exposed the whole ciliate assemblage of the UVR-flooded zone of PIB both in situ and in a more UV transparent alpine lake.

## MATERIALS AND METHODS

### Experimental sites

The ciliate assemblage used for the experiments originated from PIB, an oligo-mesotrophic lake with an area of 13.4 ha and a maximum depth of 24.6 m, which is located at 913 m above sea level in the Central Alps in Tyrol, Austria (47°11′N, 10°53′E). The lake is meromictic and usually ice-covered from December through April. The microbial food web of PIB is described in Sommaruga and Psenner (1995) and information on UV transparency is found in Laurion et al. (2000). Samples were transplanted to Gossenköllesee (GKS), a small (area: 1.7 ha) oligotrophic lake located at 2,417 m above sea level (47°13′N, 11°00′E) and beside a research station, which is easy to reach from PIB by car (ca. 1.5 h). The GKS has a maximum depth of 9.9 m and is ice-covered from November until usually June. Further information on abiotic parameters of GKS can be found in Wille et al. (1999) and on UV transparency, in Sommaruga and Augustin (2006).

### Spatial distribution of planktonic ciliates in PIB

During 2004, we assessed the seasonal and vertical distribution of the ciliate assemblage at the species level. This extensive study is not the focus of the present work and therefore, we just briefly describe the most important results relevant to this experiment. Samples were collected in the water column every 1 m from 0 to 6 m and every 3 m from 6 to 24 m depth. Water samples were taken at the deepest point of the lake from a boat with a Schindler-Patalas 5 L sampler. Subsamples of 100 ml were immediately fixed with freshly prepared Bouin's solution (5% final concentration) according to Skibbe (1994). Then, the samples were quantitatively protargol-stained and evaluated as described below.

### Lake sampling and sample transport

On August 9, 2005, plankton was collected by vertical net hauls (10-μm mesh size) at the deepest point of PIB between 06:00 and 07:00 hours local summer time. Net hauls were taken in the uppermost 7 m of the water column based on previous information on UV penetration in summer. After screening of the lake water through a 250-μm net to remove potential ciliate predators such as daphnids and copepods, ca. 12 L were placed into a clean 50 L carboy. Circa 6 L of the screened water were poured gently into a 10 L opaque carboy that was kept at ambient lake water temperature in a cooling box for transportation to GKS. During transport the screw cap of the carboy was not completely closed to allow for pressure compensation. At PIB, the remaining 6 L were kept at dim light and ambient lake water temperature until preparation of the experimental vessels similarly at both sites.

### Experimental setup and sample treatment

Subsamples of about 250 ml were poured gently into either 250-ml quartz tubes from Helios Italquartz (Milan, Italy) for exposure under UVR and PAR or into 250-ml glass bottles from Schott (Vienna, Austria) as controls. Three general triplicate setups were considered for in situ exposure in both lakes: (i) full solar radiation (UVR+PAR), (ii) PAR only by excluding most of the UVR with a vinyl chloride foil from CI Kasei Co. (Tokyo, Japan), which has a sharp cutoff (0% transmittance at 390 nm, 50% at 405 nm), and (iii) control exposed in darkness by excluding radiation with aluminum foil and two layers of black plastic foil to avoid possible reflections during exposure. Two of these setups were exposed, one just beneath the surface and one at 0.7 m in PIB or at 6 m in GKS corresponding to ca. 10% attenuation depth of UVR at 320 nm. The setups were exposed on the same day at PIB between 11:00 and 16:00 hours and at GKS from 12:00 to 17:00 hours under sunny conditions. After 5 h, 200 ml of each sample were preserved with freshly prepared Bouin's solution (5% v/v). To account for potential mortality of the ciliates during transport, triplicate T_0_-samples from the 50 L (PIB) and the 10 L carboys (GKS) were fixed before the experiment started.

In the laboratory, all ciliate samples were filtered onto cellulose-nitrate filters (0.8-μm pore size), embedded in agar and impregnated with protargol following the protocols of Skibbe (1994) and Pfister, Sonntag, and Posch (1999). From these permanent preparations, ciliates were identified following the taxonomical key of Foissner, Berger, and Schaumburg (1999 and references therein) and quantified under bright field illumination by using an Olympus BX50 microscope (Olympus, Vienna, Austria).

### Underwater irradiance

A profiling radiometer (PUV-501B, Biospherical Instruments, San Diego, CA) with nominal detection wavelengths at 305, 320, 340, and 380 nm was used to measure the attenuation of downwelling UVR in PIB on August 4, 2004 and in GKS on August 10, 2005 (these measurements are published in Sonntag et al. 2011).

### Data analysis

For detailed statistical analysis, beside the total ciliate abundance, three heterotrophic and four mixotrophic species out of ca. 65 were selected based on their dominance (abundance of >∼1,000 ind/L at T_0_ in PIB). Statistical differences in the species-specific mortality among treatments in each lake were analyzed by using a one-way ANOVA with Dunnett's post-hoc comparisons against the dark control. We compared T_0_ from PIB and GKS to test for cell loss caused during transport and experimental handling stress with a Student's *t*-test. Statistical analyses were done with the software SigmaStat 3.5 (SPSS Inc., Chicago, IL).

## RESULTS

### UV penetration

**Figure 1:**
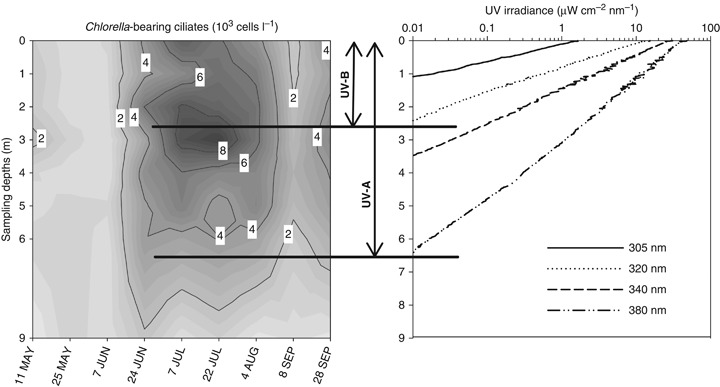
Spatial distribution of *Chlorella*-bearing ciliates in the upper 9 m of the water column in Piburgersee from May through September (left) and underwater ultraviolet radiation measured at four nominal wavelengths on August 4, 2004 (right). The horizontal upper line between graphs indicates the lower boundary of UV-B and the lower line that of UV-A penetration.

In PIB, UVR penetrated the uppermost 7 m of the water column with UV-B radiation being completely attenuated after 3 m and UV-A after ∼7 m depth (Fig. 1).

### Spatial distribution of *Chlorella*-bearing ciliates

In the 2004 study in PIB, *Chlorella*-bearing ciliates accounted for up to 52% of the total ciliate abundance between May and September in the upper 9 m of the water column (Fig. 1). The maximum abundance of 9,600 ind/L was detected at 3 m depth in July. The four most abundant mixotrophic species found during the study were the algal-bearing *Cyrtolophosis* sp. (yet undescribed species), *Pelagohalteria viridis*, and *Vorticella chlorellata*, as well as the kleptoplast-bearing *Pelagostrombidium fallax* and *Pelagostrombidium mirabile*. The latter two species were not recorded separately due to taxonomic ambiguity (see Foissner et al. 1999). The three most abundant heterotrophic species were *Rhabdoaskenasia minima, Rimostrombidium brachykinetum*/*Rimostrombidium hyalinum* (not separable during routine counting), and *Urotricha* spp. Most individuals of the latter were *Urotricha pseudofurcata*, however, some other small urotrichs were probably unintentionally included because it was impossible to clearly identify taxonomically important features in all individuals (e.g. caudal cilia or adoral organelles).

### Effect of UVR on ciliates

**Figure 2:**
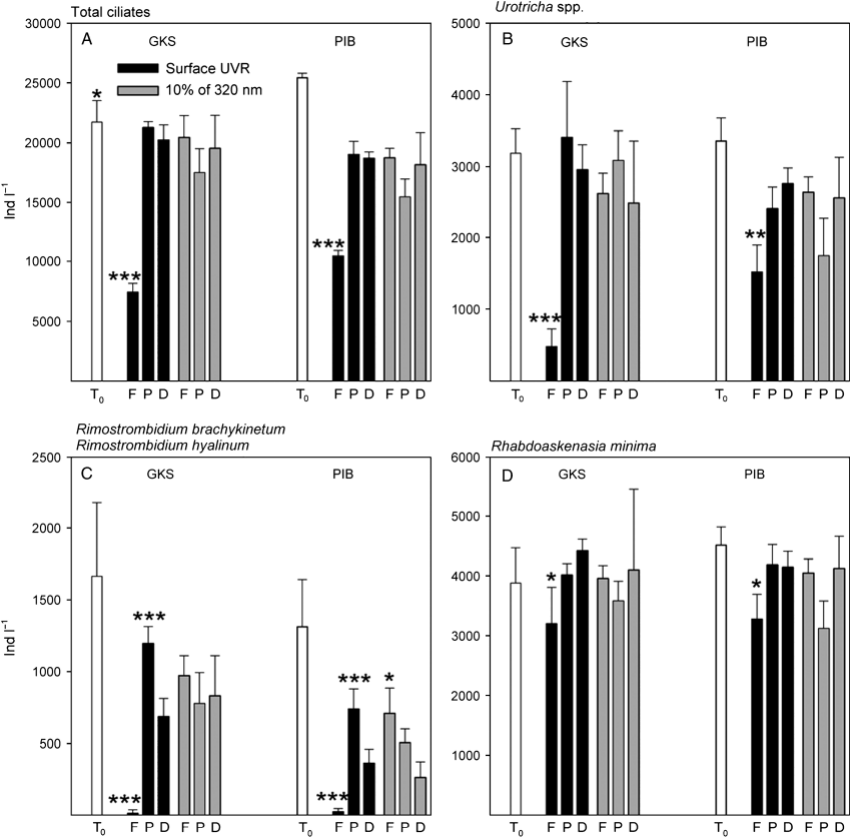
Abundance (ind/L) of total individuals (**A**) and the three heterotrophic ciliates *Urotricha* spp. (**B**), *Rimostrombidium brachykinetum/Rimostrombidium hyalinum* (**C**), and *Rhabdoaskenasia minima* (**D**) at T_0_ and after 5 h of exposure at the surface and at the depth corresponding to 10% of 320 nm in GKS and PIB. Exposure conditions were: full solar irradiation (F), photosynthetically active radiation only (P), or dark (D). Differences in the abundance at T_0_ between sites were tested by Student's *t*-test. Differences among treatments and control (D) were tested by a one-way ANOVA. Significance levels are: ^*^P≤0.05, ^**^P≤0.01, ^***^P≤0.001.

**Figure 3:**
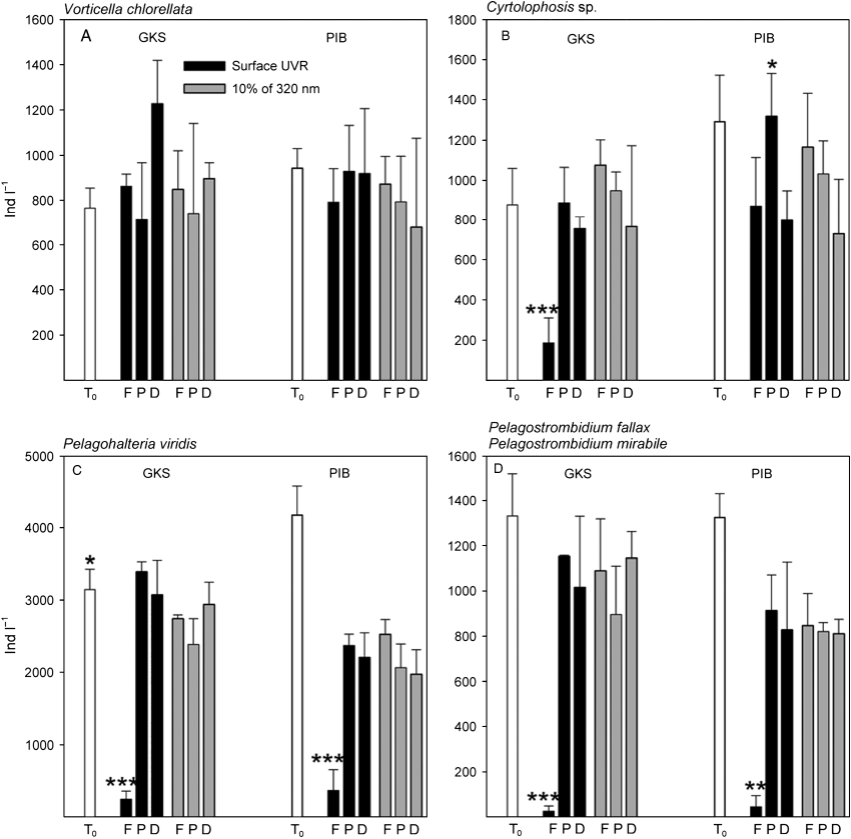
Abundance (ind/L) of the mixotrophic ciliate species *Vorticella chlorellata* (**A**), *Cyrtolophosis* sp. (**B**), *Pelagohalteria viridis* (**C**), *Pelagostrombidium fallax/Pelagostrombidium mirabile* (**D**) at T_0_ and after 5 h of exposure at the surface and at the depth corresponding to 10% of 320 nm in GKS and PIB. Exposure conditions were: full solar irradiation (F), photosynthetically active radiation only (P), or dark (D). Differences in the abundance at T_0_ between sites were tested by Student's *t-*test. Differences among treatments and control (D) were tested by a one-way ANOVA. Significance levels are: ^*^P≤0.05, ^**^P≤0.01, ^***^P≤0.001.

Overall, we detected a significant reduction in total ciliate numbers after 5 h exposure to UVR at the surface in PIB and in GKS (*P*<0.001; Fig. 2). A significant decrease in the total ciliates abundance was also detected in the T_0_ samples after their transport to GKS (*P*=0.025; Fig. 2). However, out of the selected dominant species, significantly lower cell numbers after comparison of T_0_ from PIB and GKS were found only for *P. viridis* (*P*=0.022; Fig. 3). Considering the seven selected species, a significant reduction in cell numbers after exposure to UVR at the surface in GKS was observed for all species, except for *V. chlorellata* (Fig. 2 and 3). After exposure to UVR at the surface in PIB, cell numbers significantly decreased in *P. viridis* (*P*<0.001), *P. fallax/P. mirabile* (*P*=0.003), *R. minima* (*P*=0.029), *R. brachykinetum/R. hyalinum* (*P*<0.001), and *Urotricha* spp. (*P*=0.006). At the 10% attenuation depth, no significant reduction in cell numbers was observed in either lake.

## DISCUSSION

### Mixotrophic species

Overall, we could not find a general trend of higher UVR resistance in mixotrophic than in heterotrophic ciliates, though the *Chlorella*-bearing species *V. chlorellata* was able to withstand the UVR at the surface in both lakes (Fig. 3). Interestingly, this species is known to possess MAAs (Sonntag et al. 2007). These secondary metabolites are effective photoprotective compounds absorbing UVR between 309 and 362 nm (Shick and Dunlap 2002; Volkmann et al. 2006). The presence of the compounds in the cytoplasm provides planktonic organisms with a “broad-band” UV-filter and protection from harmful wavelengths (Garcia-Pichel 1994). The origin of these secondary metabolites in *Chlorella*-bearing ciliates is their algal symbionts and previous studies have shown, for example, that in the *Askenasia chlorelligera* population from GKS, MAAs concentrations are 10 times higher than in several *Chlorella*-bearing species from PIB indicating their adaptive response to higher UV levels (Sonntag et al. 2007; Summerer et al. 2008). Interestingly, the *A. chlorelligera* population from PIB apparently lacks MAAs probably as consequence of the lower UVR levels this species experiences in this lake (Summerer et al. 2008). Apparently, the population of *A. chlorelligera* from GKS can withstand the high UVR conditions found at the surface, even if this species is not fully packed with *Chlorella* (Sonntag et al. 2011).

**Figure 4:**
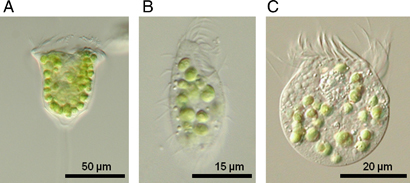
Digital images of three living *Chlorella*-bearing ciliates, i.e. *Vorticella chlorellata* (**A**), *Cyrtolophosis* sp. (**B**) and *Pelagohalteria viridis* (**C**).

The fact that *V. chlorellata* was unaffected by UVR in both lakes (Fig. 3) is coincident with the observation that their maximum population abundance in PIB was in July at the surface, and suggests this species is well adapted to high solar radiation levels. This is also in agreement with the finding that one of the highest MAA concentrations among ciliates from PIB was found in *V. chlorellata* (Sonntag et al. 2007). Besides the presence of MAAs, this species has densely packed *Chlorella* symbionts in the cytoplasm (representing up to 95% of the ciliate cell volume based on a cell volume for *Chlorella* of 65 μm^3^) that provide additional self-shading by cell matter absorption (Fig. 4). In this latter aspect, *V. chlorellata* is similar to *P. bursaria* (Summerer et al. 2009). The screening factor of the algal symbionts can be estimated after the optical model of Garcia-Pichel (1994). For example, Summerer et al. (2009) calculated that self-shading at 320 nm of one *Chlorella*-layer in a ciliate was 35% and that five layers and more as observed in *P. bursaria* provide >98% shading. Taken together, these findings support our result that *V. chlorellata* was resistant against UVR not only in its original habitat, but also in GKS.

*Cyrtolophosis* sp. is a yet undescribed colpodid species that bears algal symbionts (Fig. 4). This tiny ciliate (22 × 9 μm) is a commonly occurring species in PIB during summer in the uppermost 9 m with its maximum abundance found at 3 m depth in September. In July, maximum numbers are found at the surface suggesting that the ciliate needs to cope with high UVR levels. Unfortunately, the presence of MAAs in *Cyrtolophosis* sp. is unknown. In this small species, only between six and 12 algal symbionts (i.e. volume of the ciliate occupied by algal symbionts: 44–89%) are found, suggesting that self-shading of UVR by *Chlorella* is probably not very efficient (Fig. 4). Nevertheless, though the elevated UVR at the surface in GKS caused a significant cell loss in *Cyrtolophosis* sp., the species seems to be well adapted to the UVR regime in its original lake, as no significant mortality was found (Fig. 3).

*Pelagohalteria viridis* is an oligotrich ciliate usually found mainly in the uppermost 9 m of the water column of PIB with maximum numbers detected at around 3 m depth in July. The species was highly sensitive to UVR at the surface of both lakes (Fig. 3). This is coincident with the observation that *P. viridis* is found below the lower boundary of the UV-B penetration in PIB (data not shown). Though *P. viridis* is sometimes densely packed with algal symbionts, they have a particular arrangement (Fig. 4). In this small species (20–30 μm diameter), the algal symbionts are arranged in two clumps right and left of the pharynx occupying a volume between 31% and 64% (Foissner et al. 1999). Assuming that an algal symbiont has a diameter of about 3–5 μm, the small cell size of *P. viridis* allows for a maximum of two to three symbiont layers around the nuclei. According to the calculations of Summerer et al. (2009), two symbiont layers would block <75% of the UVR at 320 nm. Hence, as *P. viridis* has not only jumping, but also rotating movements, a direct impact of UVR onto its nuclei might be partially avoided. Nevertheless, remaining in water layers where UV-B is not present probably enhances its overall survival.

Finally, *P. fallax* and *P. mirabile* are two oligotrich species that have kleptoplasts. Cell numbers were significantly reduced after exposure to UVR at the surface in both lakes suggesting that kleptoplasts are not efficient in offering photoprotection. The high sensitivity of these species is coincident with the finding that at times of the highest incident solar radiation, they are only present in low numbers (data not shown). This seasonal pattern is similar to that found in Lake Constance, Mondsee, and Traunsee (Müller and Wünsch 1999; Müller, Stadler, and Weisse 2002; Sonntag et al. 2006). In the first two lakes, two maxima in abundance, one in spring and one in autumn are observed, while in Traunsee only one maximum in autumn is detected. It is known that strombidiids form cysts, especially after reaching their maximum seasonal abundance, and that temperature and probably also food triggered encystment and excystment (Müller 2002; Müller and Wünsch 1999; Müller et al. 2002). Considering the high sensitivity of this species in our experiments, we suggest that encystment of *P. fallax/P. mirabile* after the spring abundance maximum could also be triggered by UVR.

### Heterotrophic species

All heterotrophic ciliate species were significantly affected by UVR when exposed at the surface in both lakes, but not at the 10% attenuation depth (Fig. 2).

At times of the phytoplankton spring bloom, small oligotrich and prostomatid ciliates such as *R. brachykinetum/R. hyalinum, Urotricha* spp. or *B. planctonicum* are predominant (e.g. Müller et al. 1991; Sonntag et al. 2006). In fact, these species can be responsible for the early clear-water phase beside zooplankton (Sommaruga and Psenner 1993; Tirok and Gaedke 2006). Though, these ciliates are known to colonize the “surface layers”, in our experiment, they were highly sensitive to UVR at the surface of both lakes (Fig. 2). Considering the vertical distribution from May through September 2004 of *Urotricha* spp., *R. brachykinetum/R. hyalinum*, and *R. minima* (data not shown), maximum numbers were never detected in the uppermost meter. This characteristic deep distribution suggests that these species are not exposed to high UV-B radiation levels in their natural habitat. By contrast, species such as *Urotricha* cf. *castalia* and *Urotricha pelagica* from GKS are able to withstand high incident UVR levels not only during short-term exposure, but also over several days (Sommaruga et al. 1999; Sonntag et al. 2011).

Another mechanism to reduce cell mortality is the activation of photoenzymatic repair by UV-A and visible light. For example, in the heterotrophic species *Glaucoma* sp., Sanders et al. (2005) observed that photorepair was an efficient photoprotective strategy to cope with damages caused by UV-B. Sanders et al. (2005) further found that the efficiency of photoenzymatic repair was strongly temperature-dependent in this species. For example, it was more efficient at 20 °C than at lower or higher temperatures of 10 °C, 15 °C, or 25 °C. This optimum temperature coincided with that where *Glaucoma* sp. attained its maximum abundance (Sanders et al. 2005). In our study, photoenzymatic repair, if present, was not sufficient to avoid mortality of most species when exposed at the surface in PIB, even if water temperature (21 °C) was probably within the optimum range of most ciliate species. On the other hand, the colder water (surface: 10 °C) in GKS may have exacerbated the mortality by UVR, by reducing the photoenzymatic repair activity.

### Conclusions

Our results show that no generalization can be made for *Chlorella*-bearing ciliates regarding their expected higher tolerance of UVR. Further, our study stresses the importance of testing the sensitivity to UVR of natural mixotrophic ciliate assemblages that include species with very different degree of algal self-shading. On the other hand, the results clearly indicate that species such as *V. chlorellata* that can attain high self-shading by *Chlorella* and possess MAAs are resistant to UVR and are probably able to better compete with other less tolerant species at the surface. Our study also illustrates that the information gathered on depth preference of ciliate species over time and thus, on their average exposure history is also an important factor to understand their UV sensitivity in these experiments. Finally, this study shows that exposure at the 10% attenuation depth, which is sometimes assumed to represent the lower boundary where potential damage by UVR can be expected, did not affect this freshwater ciliate assemblage.
